# Impact of mental illness on end‐of‐life emergency department use in elderly patients with gastrointestinal malignancies

**DOI:** 10.1002/cam4.3792

**Published:** 2021-02-23

**Authors:** Mehr Kashyap, Jeremy P. Harris, Daniel T. Chang, Erqi L. Pollom

**Affiliations:** ^1^ Department of Radiation Oncology Stanford University Stanford California USA; ^2^ Stanford University School of Medicine Stanford University Stanford California USA; ^3^ Department of Radiation Oncology University of California Irvine, Orange California USA

**Keywords:** cancer management, digestive cancer, quality of life, SEER‐Medicare

## Abstract

**Background:**

Elderly patients with gastrointestinal cancer and mental illness have significant comorbidities that can impact the quality of their care. We investigated the relationship between mental illness and frequent emergency department (ED) use in the last month of life, an indicator for poor end‐of‐life care quality, among elderly patients with gastrointestinal cancers.

**Methods:**

We used SEER‐Medicare data to identify decedents with gastrointestinal cancers who were diagnosed between 2004 and 2013 and were at least 66 years old at time of diagnosis (median age: 80 years, range: 66–117 years). We evaluated the association between having a diagnosis of depression, bipolar disorders, psychotic disorders, anxiety, dementia, and/or substance use disorders and ED use in the last 30 days of life using logistic regression models.

**Results:**

Of 160,367 patients included, 54,661 (34.1%) had a mental illness diagnosis between one year prior to cancer diagnosis and death. Patients with mental illness were more likely to have > 1 ED visit in the last 30 days of life (15.6% vs. 13.3%, *p* < 0.01). ED use was highest among patients with substance use (17.7%), bipolar (16.5%), and anxiety disorders (16.4%). Patients with mental illness who were male, younger, non‐white, residing in lower income areas, and with higher comorbidity were more likely to have multiple end‐of‐life ED visits. Patients who received outpatient treatment from a mental health professional were less likely to have multiple end‐of‐life ED visits (adjusted odds ratio 0.82, 95% confidence interval 0.78–0.87).

**Conclusions:**

In elderly patients with gastrointestinal cancers, mental illness is associated with having multiple end‐of‐life ED visits. Increasing access to mental health services may improve quality of end‐of‐life care in this vulnerable population.

## Graphical abstract

Frequent end‐of‐life emergency department (ED) use is an indicator for poor quality of care. Elderly patients with gastrointestinal malignancies and comorbid mental illness are more likely to have multiple ED visits at the end of life; however, receipt of early mental health services mitigates this risk.

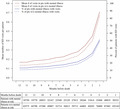

## 1 INTRODUCTION

Patients with cancer frequently visit the emergency department (ED) at the end of life.^1^, ^2^ More than 10% of elderly patients use the ED multiple times in the last month of life, and this proportion has been increasing despite attempts to reduce unnecessary visits.^3^, ^4^ ED use and subsequent hospitalizations can be disruptive for patients and contribute significantly to the rising cost of cancer care.^5^, ^6^, ^7^ In addition, these visits often result from a lack of sufficient symptom management and palliative care.^5^, ^8^ Thus, limiting the proportion of patients with frequent end‐of‐life ED visits is a well‐recognized component of delivering high‐quality and cost‐effective care.^9^

While socioeconomic and demographic factors have been shown to influence end‐of‐life ED use, the impact of clinical factors such as mental illness is poorly understood.^10^ Cancer patients have a high rate of mental illness, which is linked to disparities in care.^11^, ^12^ For instance, patients with mental illness are screened for cancer less frequently, diagnosed at a later stage, receive non‐definitive treatment more commonly, and have higher mortality rates.^13^, ^14^, ^15^, ^16^

We investigated the impact of mental illness on end‐of‐life ED use among elderly patients with gastrointestinal cancers since mental illness is prevalent in this population and it negatively influences patient care.^15^, ^17^, ^18^ Those with gastrointestinal cancers also have a unique set of symptoms and needs, with common complications including bowel and biliary obstruction, gastrointestinal bleeding, and pain. As a result, patients with gastrointestinal cancers appear to visit the ED more frequently than those with other types of cancers.^19^ Therefore, defining the relationship between mental illness and end‐of‐life ED use in this population is vital for both identifying disparities in care and establishing effective interventions for high‐risk patients.

## 2 MATERIALS AND METHODS

### 2.1 Data source

We used the Surveillance, Epidemiology and End Results (SEER)‐Medicare linked database, which provides longitudinal clinical information on Medicare beneficiaries with cancer by linking cancer incidence and survival data from population‐based SEER cancer registries with insurance claims data from Medicare.^20^ Each cancer registry collects information about patient demographics, cancer diagnosis, tumor characteristics, and initial course of treatment. Medicare claims data are linked on the patient level and provide detailed information about hospital, physician, and outpatient health services for all patients included in the database. This study received institutional review board exemption from the Stanford University School of Medicine.

### 2.2 Patient selection

**FIGURE 1:**
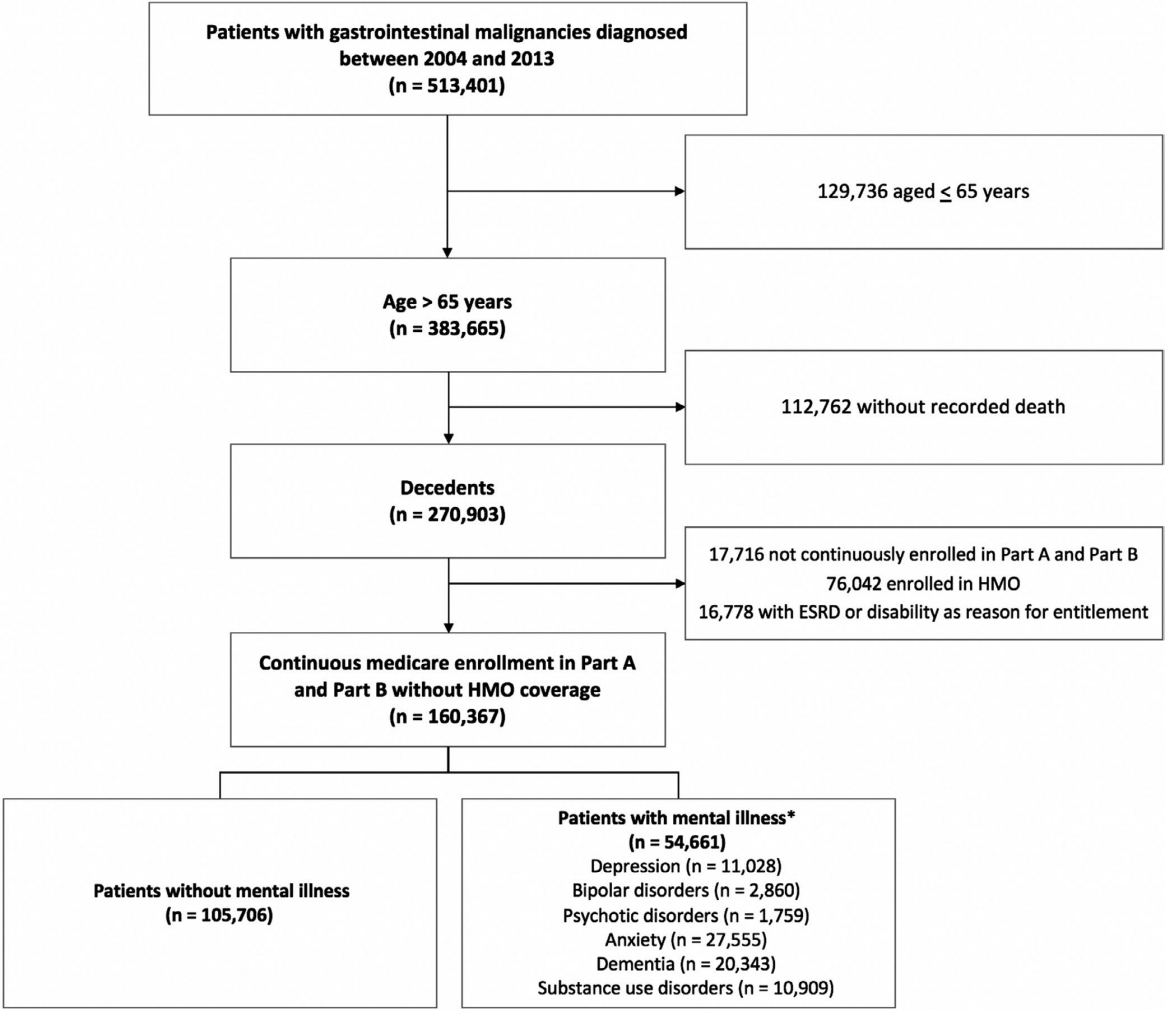
Cohort selection. *Patients with multiple mental illnesses were included in all applicable categories

We identified patients with a first gastrointestinal malignancy diagnosed between 2004 and 2013 (Figure 1). These included individuals with primary colorectal, pancreatic, gastric, hepatic, biliary, esophageal, small bowel, anal, and other (peritoneal, retroperitoneal, or unspecified) cancers. Patients included were at least 66 years old at time of cancer diagnosis, had a recorded death date, and were continuously enrolled in Medicare Parts A and B from one year prior to cancer diagnosis until death. We excluded patients who were members of health maintenance organizations or were eligible for insurance due to disability or end stage renal disease.

### 2.3 Mental illness identification

Patients were classified as having a mental illness based on the presence of relevant International Classification of Diseases, Ninth Revision, Clinical Modification (ICD‐9‐CM) codes in any inpatient or outpatient claims between one year prior to cancer diagnosis and death. We included patients diagnosed with mental illness prior to cancer diagnosis since there is growing evidence that having a pre‐existing mental illness can impact the quality of cancer care patients receive, which, in turn, may impact quality of end‐of‐life care.^16^ We identified patients with depression, bipolar disorders, psychotic disorders, anxiety, dementia, and substance use disorders (Table S1). We did not include patients with personality disorders in our analysis since we identified fewer than 150 patients with these disorders that met inclusion criteria. Patients with more than one mental illness were included in all applicable categories.

### 2.4 Patient characteristics

We extracted patient age, sex, race, Hispanic ethnicity, marital status, and census tract‐level median household income from SEER. Patient race was defined independently of Hispanic ethnicity^21^ and included the following categories: White, Black, Asian or Pacific Islander, American Indian, or Alaska Native. Primary site was determined using International Classification of Diseases for Oncology Third Edition (ICD‐O‐3) topography codes. We also classified cancer stage as local, regional, or distant based on the summary stage reported in SEER.^22^ Comorbidity burden was estimated from insurance claims in the year prior to cancer diagnosis using the Klabunde modification of the Charlson Comorbidity Index.^23^, ^24^

### 2.5 Identification of emergency department visits

ED visits were identified through insurance claims by using a combination of Healthcare Common Procedure Coding System (HCPCS), Berenson‐Eggers Type of Service (BETOS), and inpatient admission codes.^3^ Specific codes are presented in Table S2. We identified ED visits made by all patients within the last year of life. We also specifically determined whether patients visited the ED multiple times in the last 30 days of life, an indicator for poor quality end‐of‐life cancer care.^9^

To identify reasons for ED visits, we extracted the first ICD‐9‐CM diagnosis code from all relevant claims and reassigned these codes into clinically meaningful categories using the Agency for Healthcare Research and Quality (AHRQ) Clinical Classification Software.

### 2.6 Professional management of patients with mental illness

We classified patients as having received professional management for mental illness if they had at least one provider claim for a mental health service within a year of their first diagnosed mental illness. Table S3 includes the specific HCPCS, Center for Medicare Services (CMS) specialty, and revenue center codes that were used.

### 2.7 Statistical analysis

We compared baseline patient characteristics between those with and without mental illness using Pearson's chi‐squared test. We evaluated the association between various mental illnesses and multiple ED visits within the last 30 days of life using multivariable logistic regression models. We included all extracted patient characteristics as model covariates to mitigate the effect of confounding factors.

The significance level for all tests was set to 0.05. All statistical analyses were performed using R (version 3.4.3, R Foundation for Statistical Computing, Vienna, Austria).

## 3 RESULTS

In total, 160,367 patients with gastrointestinal cancers met inclusion criteria, of whom 54,661 (34.1%) had at least one diagnosed mental illness between one year prior to cancer diagnosis and death. The cohort of patients with mental illness was more highly represented by individuals who were at least 85 years old, women, white, and unmarried, had colorectal or anal cancer, earlier stage disease, and higher comorbidity burden (Table 1).

**TABLE 1:** Demographic and clinical characteristics of patients with gastrointestinal cancers by mental illness status

Characteristics	No Mental Illness (*n* = 105,706, 65.9%)	Any Mental Illness (*n* = 54,661, 34.1%)	*p*
disease site (%)			
Colorectal	42,702 (40.4)	27,584 (50.5)	<0.001
Pancreas	25,912 (24.5)	9,544 (17.5)	
Stomach	10,746 (10.2)	4,839 (8.9)	
Liver and intrahepatic bile ducts	8,803 (8.3)	4,201 (7.7)	
Esophagus	6,743 (6.4)	3,236 (5.9)	
Gallbladder and extrahepatic bile ducts	5,734 (5.4)	2,421 (4.4)	
Small intestine	1,818 (1.7)	994 (1.8)	
Anus	920 (0.9)	766 (1.4)	
Other	2,328 (2.2)	1,076 (2.0)	
Age (%)			
85+	28,214 (26.7)	15,257 (27.9)	<0.001
75–84	47,301 (44.8)	23,878 (43.7)	
66–74	30,039 (28.5)	15,464 (28.3)	
Sex (%)			
Female	51,168 (48.4)	30,368 (55.6)	<0.001
Male	54,538 (51.6)	24,293 (44.4)	
Race (%)			
White	88,800 (84.0)	47,208 (86.4)	<0.001
American Indian/Alaska Native	485 (0.5)	207 (0.4)	
Asian or Pacific Islander	7,166 (6.8)	2,371 (4.3)	
Black	9,067 (8.6)	4,791 (8.8)	
Unknown	188 (0.2)	84 (0.2)	
Ethnicity (%)			
Non‐Hispanic	99,291 (93.9)	51,234 (93.7)	0.115
Hispanic	6,415 (6.1)	3,427 (6.3)	
Marital status (%)			
Single	7,812 (7.4)	4,972 (9.1)	<0.001
Divorced/Separated	7,154 (6.8)	4,553 (8.3)	
Married/Domestic Partner	51,411 (48.6)	22,767 (41.7)	
Widowed	34,841 (33.0)	19,745 (36.1)	
Unknown	4,488 (4.2)	2,624 (4.8)	
Household income by area (%)			
Top quantile	23,907 (22.6)	12,518 (22.9)	<0.001*
2nd quantile	23,960 (22.7)	12,470 (22.8)	
3rd quantile	23,903 (22.6)	12,527 (22.9)	
Bottom quantile	23,935 (22.6)	12,491 (22.9)	
Unknown	10,001 (9.5)	4,655 (8.5)	
Stage (%)			
Local	22,212 (21.0)	16,461 (30.1)	<0.001
Regional	29,343 (27.8)	16,576 (30.3)	
Distant	39,475 (37.3)	13,883 (25.4)	
Unknown	14,676 (13.9)	7,741 (14.2)	
Charlson comorbidity index (%)			
0	48,672 (46.0)	19,798 (36.2)	<0.001
1	26,793 (25.3)	13,738 (25.1)	
2	14,014 (13.3)	8,511 (15.6)	
3+	16,227 (15.4)	12,614 (23.1)	

* *p*‐value was 0.97 when unknown values were excluded.

### 3.1 End‐of‐life ED use and associated risk factors

**FIGURE 2:**
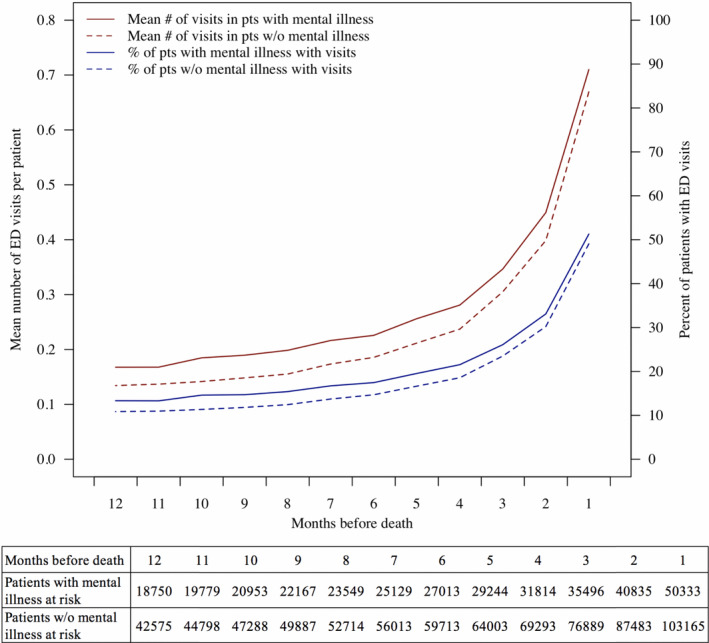
ED visits in the last year of life. Abbreviations: ED, emergency department. pts, patients. w/o, without

ED use consistently increased throughout the last year of life (Figure 2). The sharpest increase in use occurred in the last month of life, during which 51.3% of patients with and 49.1% of patients without mental illness visited the ED at least once.

Patients with mental illness were more likely to visit the ED multiple times in the month before death than those without mental illness (15.6% vs. 13.3%, *p* < 0.01; Table 2). In particular, we observed higher odds of multiple ED visits in the last 30 days of life in patients with anxiety (adjusted odds ratio [aOR] 1.26, 95% confidence interval [CI] 1.21–1.31), substance use (aOR 1.18, 95% CI 1.12–1.24), and bipolar (aOR 1.12, 95% CI 1.01–1.24) disorders. We also examined risk factors associated with multiple end‐of‐life ED visits among patients with mental illness (Table 3). These included being male (aOR 1.17, 95% CI 1.11 – 1.23), younger (aOR 1.21, 95% CI 1.13 – 1.30), black (aOR 1.51, 95% CI 1.40 – 1.64), Asian or Pacific Islander (aOR 1.15, 95% CI 1.03 – 1.29), and Hispanic (aOR 1.20, 95% CI 1.10 – 1.32). Risk factors also included living in a lower income census tract (aOR 1.10, 95% CI 1.03 – 1.18 for third income quantile) and having a higher Charlson comorbidity index (aOR 1.26, 95% CI 1.18 – 1.34 for score ≥ 3). Furthermore, end‐of‐life ED visits were associated with pancreatic (aOR 1.10, 95% CI 1.03 – 1.18), hepatic (aOR 1.17, 95% CI 1.07 – 1.28), esophageal (aOR 1.17, 95% CI 1.06 – 1.29), biliary (aOR 1.16, 95% CI 1.03 – 1.30), small bowel cancer (aOR 1.22, 95% CI 1.03 – 1.44), and other gastrointestinal cancers (peritoneal, retroperitoneal, or unspecified; aOR 1.19, 95% CI 1.01–1.41) compared to colorectal cancer. Regional disease was associated with lower odds of having multiple end‐of‐life ED visits compared to local disease (aOR 0.91, 95% CI 0.86 – 0.97).

**TABLE 2:** Impact of mental illness on having multiple ED visits in the last 30 days of life

Risk factor	>1 ED visit in last 30 days of life		
	No. of patients (%)	Unadjusted OR (95% CI)	Adjusted OR (95% CI)
No mental illness (n = 105,706)	14,100 (13.3)	Reference	Reference
Any mental illness (n = 54,661)	8,531 (15.6)	1.20 (1.17–1.24)	1.20 (1.16–1.24)
Depression (n = 11,028)	1,635 (14.8)	1.06 (1.01–1.12)	0.97 (0.92–1.03)
Bipolar disorders (n = 2,860)	473 (16.5)	1.21 (1.1–1.34)	1.12 (1.01–1.24)
Psychotic disorders (n = 1,759)	274 (15.6)	1.12 (0.99–1.28)	0.98 (0.85–1.12)
Anxiety (n = 27,555)	4,522 (16.4)	1.24 (1.2–1.29)	1.26 (1.21–1.31)
Dementia (n = 20,343)	2,903 (14.2)	1.02 (0.97–1.06)	1.02 (0.97–1.06)
Substance use disorders (n = 10,909)	1,928 (17.7)	1.34 (1.27–1.41)	1.18 (1.12–1.24)

Abbreviations: ED, emergency department; OR, odds ratio.

**TABLE 3:** Risk factors associated with having multiple ED visits in the last 30 days of life among patients with mental illness

Risk factor		>1 ED visit in last 30 days of life OR (95% CI)
Disease site	Colorectal	1.0 (Ref)
	Pancreas	1.1 (1.03–1.18)
	Stomach	1.06 (0.97–1.16)
	Liver and intrahepatic bile ducts	1.17 (1.07–1.28)
	Esophagus	1.17 (1.06–1.29)
	Gallbladder and extrahepatic bile ducts	1.16 (1.03–1.3)
	Small intestine	1.22 (1.03–1.44)
	Anus	1.08 (0.87–1.31)
	Other	1.19 (1.01–1.41)
Age	85+	1.0 (Ref)
	75–84	1.14 (1.07–1.21)
	66–74	1.21 (1.13–1.3)
Sex	Female	1.0 (Ref)
	Male	1.17 (1.11–1.23)
Race	White	1.0 (Ref)
	American Indian/Alaska Native	0.99 (0.6–1.42)
	Asian or Pacific Islander	1.15 (1.03–1.29)
	Black	1.51 (1.4–1.64)
	Unknown	0.81 (0.4–1.47)
Ethnicity	Non‐Hispanic	1.0 (Ref)
	Hispanic	1.2 (1.1–1.32)
Marital status	Single	1.0 (Ref)
	Divorced/Separated	0.98 (0.88–1.09)
	Married/Domestic Partner	1.06 (0.98–1.16)
	Widowed	1.04 (0.96–1.14)
	Unknown	1.12 (0.99–1.28)
Household income by area	Top quantile	1.0 (Ref)
	2nd quantile	1.04 (0.97–1.12)
	3rd quantile	1.1 (1.03–1.18)
	Bottom quantile	1.07 (1–1.15)
	Unknown	0.96 (0.87–1.06)
Stage	Local	1.0 (Ref)
	Regional	0.91 (0.86–0.97)
	Distant	0.98 (0.92–1.05)
	Unknown	0.71 (0.66–0.78)
Charlson comorbidity index	0	1.0 (Ref)
	1	1.1 (1.04–1.17)
	2	1.1 (1.02–1.18)
	3+	1.26 (1.18–1.34)
Mental illness	Depression	0.99 (0.91–1.08)
	Bipolar disorders	1.14 (1.02–1.28)
	Psychotic disorders	1.01 (0.88–1.16)
	Anxiety	1.23 (1.13–1.34)
	Dementia	1 (0.92–1.1)
	Substance use disorders	1.15 (1.05–1.26)
	Multiple (vs. single)	0.94 (0.85–1.05)

Abbreviations: ED, emergency department; OR, odds ratio.

### 3.2 Common reasons for end‐of‐life ED visits

Abdominal pain was the most common reason for ED visits in the last month of life, comprising 7.5% of all visits for patients with mental illness and 9.6% for those without (Table 4). Lower respiratory disease, malaise and fatigue, hypovolemia, septicemia, pneumonia, gastrointestinal hemorrhage, and liver disease were other common diagnoses shared between both groups.

**TABLE 4:** Most common reasons for ED visits in the last 30 days of life

Primary Diagnosis for ED Visit	Percent of all ED Visits in Cohort
Any Mental Illness	
Abdominal pain	7.5%
Lower respiratory disease^a^	5.5%
Pneumonia (except that caused by TB or STD)	4.6%
Septicemia (except in labor)	4.3%
Hypovolemia	4.2%
Malaise and fatigue	3.9%
Hemorrhage of gastrointestinal tract	2.7%
Liver diseases^a^	2.5%
Urinary tract infections	2.1%
Congestive heart failure (non‐hypertensive)	2.1%
No Mental Illness	
Abdominal pain	9.6%
Lower respiratory disease^a^	5.3%
Malaise and fatigue	4.6%
Hypovolemia	4.5%
Septicemia (except in labor)	3.3%
Pneumonia (except that caused by TB or STD)	3.3%
Hemorrhage of gastrointestinal tract	3.0%
Liver diseases^a^	2.8%
Nausea and vomiting	2.5%
Intestinal obstruction without hernia	2.4%

Abbreviations: ED, emergency department.

^a^ other and unspecified type.

### 3.3 Impact of professional management of mental illness on end‐of‐life ED use

Patients who received professional management for mental illness were less likely than those who did not to have visited the ED multiple times in the last 30 days of life (13.7% vs 16.3%, *p* < 0.01; Table 5). Receiving mental health services was significantly associated with reduced odds of multiple end‐of‐life ED visits (aOR 0.82, 95% CI 0.78–0.87).

**TABLE 5:** Impact of professional management of mental illness on having multiple ED visits in the last 30 days of life

Professional management for mental illness	>1 ED visit in last 30 days of life (n = 8,531)		
	No. of patients (%)	Unadjusted OR (95% CI)	Adjusted OR (95% CI)
Received treatment (n = 14,885)	2,044 (13.7)	0.82 (0.77–0.86)	0.82 (0.78–0.87)
Did not receive treatment (n = 39,776)	6,487 (16.3)	Reference	Reference

Abbreviations: ED, emergency department; OR, odds ratio.

## 4 DISCUSSION

Elderly patients with gastrointestinal cancer and mental illness have a high degree of comorbidity that can impact the quality of their cancer care and compromise outcomes. In our population‐based analysis, we found that having a mental illness was associated with receipt of poorer quality end‐of‐life care among these patients. Specifically, patients with mental illness were more likely to use the ED multiple times in their last month of life. Further, we found that receipt of early mental health services mitigates this risk, highlighting the importance of effective mental health support and interventions in this population.

End‐of‐life ED visits can be disruptive for patients during a time when most patients prefer home‐based care and less aggressive interventions.^25^, ^26^ There is increasing recognition that these visits signal poor quality cancer care. For instance, the American Society of Clinical Oncology supports a quality measure aimed at reducing the proportion of patients with multiple ED visits in the last 30 days of life.^9^ From a health care system perspective, these visits and subsequent hospitalizations significantly contribute to cancer costs, as the majority of health care expenditures in the last month of life stem from acute inpatient care.^7^ Therefore, addressing disparities in end‐of‐life care, such as those that exist for patients with mental illness, may improve care quality and reduce costs.

We suspect that mental illness impacts end‐of‐life ED use through several different biological, social, and behavioral pathways. For instance, having a mental illness influences which treatments patients receive, whether through patient or physician choice, and this has downstream consequences on health.^13^, ^15^ Patients with mental illness also frequently have poor social support, which makes it difficult to navigate the healthcare system and adhere to treatments and medical appointments.^27^, ^28^ Furthermore, psychological distress can complicate medical decision‐making and lead to receiving high intensity end‐of‐life interventions.^29^ Finally, patients with mental illness are less likely to participate in advance care planning, which is important for clarifying preferences about end‐of‐life care.^30^

Specifically, we found that anxiety, bipolar, and substance use disorders were associated with multiple ED visits in the last month of life. In contrast, depression, psychotic disorders, and dementia were not associated with frequent ED use at this stage. This variability in impact is likely related to multiple factors. Patients with particular mental illnesses experience unique barriers to accessing care and often respond to symptoms such as pain differently, which can influence subsequent use of emergency services.^31^ For instance, increased anxiety appears to be closely linked with decreased pain tolerance,^32^ and abdominal pain was the most common reason for ED visits in our study. Further studies are needed to better understand differences between these disorders with respect to end‐of‐life care; however, our study specifies a subset of cancer patients with mental illness who are at highest risk for ED use at the end of life and thus require greater attention from health care providers.

Additional factors associated with ED use are race, gender, socioeconomic status and comorbidity burden. Black patients were more likely to have multiple ED visits prior to death. Asian, Pacific Islander, and Hispanic patients also had a higher risk of frequent visits. This may be partly driven by patient preference; however, it may also reflect structural disparities, including access to palliative services.^33^, ^34^ Given the strong evidence for racial and ethnic disparities in end‐of‐life cancer care,^35^, ^36^ minority patients with mental illness represent a particularly high‐risk group. Younger age, male sex, living in a lower income area, and higher comorbidity were also associated with having multiple end‐of‐life ED visits among those with mental illness, which in consistent with previous studies.^10^

Furthermore, those with pancreatic, hepatic, esophageal, biliary, and small bowel cancer were more likely to visit the ED compared to patients with colorectal cancer. This may be related to the aggressiveness of various cancer subtypes, as shorter post‐diagnosis survival time is associated with more intense end‐of‐life care^37^; however, we also found that patients with regional disease are less likely to use the ED frequently at the end of life than those with local disease. Thus, it appears that the relationship between aggressiveness of disease and end‐of‐life care is complex and likely involves various factors such as post‐diagnosis survival time and symptom burden. These results stratify risk among cancer patients with mental illness and should inform interventions in this population.

Most encouragingly, we found that patients who receive mental health services soon after being diagnosed with a mental illness are less likely to require ED use prior to death. This is a promising avenue for intervention since cancer patients with mental illness have a low rate of accessing mental health services.^12^ Thus, it is important for oncologists to partner with mental health providers to better screen for and address mental illness among patients with cancer.

The most common reasons that patients with mental illness visited the ED in our analysis were abdominal pain, respiratory distress, infection, hypovolemia, and malaise and fatigue. These results signal additional opportunities to intervene, as visits made for poorly controlled symptoms are likely preventable with effective outpatient strategies.^2^, ^8^ In particular, expanding access to palliative and supportive services may help reduce unnecessary end‐of‐life ED visits.^10^ Furthermore, early palliative care has also been shown to improve quality of life, reduce caregiver burden and distress, decrease aggressive end‐of‐life interventions, and even increase survival.^38^, ^39^, ^40^

This study has several important limitations. First, we used administrative data to conduct our analysis. In particular, we used diagnosis codes in insurance claims to detect mental illness, which can lead to patient misclassification.^41^ Second, this is a retrospective analysis and is therefore susceptible to selection bias and confounding; however, this study design is well‐suited for assessing patterns of end‐of‐life care across populations as it allows for efficient identification of terminally ill patients.^42^ Third, we did not have access to important factors that influence end‐of‐life care, such as patient preferences, advance care planning, and social support in our data. Finally, our cohort consisted of Medicare beneficiaries with gastrointestinal cancers, which limits the generalizability of our study.

## 5 CONCLUSIONS

Elderly patients with gastrointestinal cancers and comorbid mental illness have an increased risk of visiting the ED multiple times at the end of life. Improving access to mental health services reduces this risk and is therefore an important component of delivering high‐quality care. Future studies should investigate barriers to accessing mental health services among this population.

## ETHICAL APPROVAL STATEMENT

This study received institutional review board exemption from the Stanford University School of Medicine.

## CONFLICT OF INTEREST

Erqi Pollom has received honorarium from Accuray.

## AUTHOR CONTRIBUTIONS

**Mehr Kashyap:** Conceptualization, data curation, methodology, investigation, writing‐original draft, and writing‐reviewing and editing. **Jeremy P. Harris:** Data curation, and writing‐reviewing and editing. **Daniel T. Chang**: Conceptualization, methodology, investigation, and writing‐reviewing and editing. **Erqi L. Pollom**: Conceptualization, methodology, investigation, writing‐original draft, and writing‐reviewing and editing.

## Supporting information

Table S1‐S3

## Data Availability

The data that support the findings of this study are available through the Surveillance, Epidemiology and End Results (SEER)‐Medicare linked database. Restrictions apply to the availability of these data, which were used under license for this study. Data are available at https://healthcaredelivery.cancer.gov/seermedicare/.
