# Supplementary material for: Impact of mental illness on end‐of‐life emergency department use in elderly patients with gastrointestinal malignancies
Source: Cancer Med. 2021 Feb 23;10(6):2035–44. doi: 10.1002/cam4.3792 (PMC7957203; doi:10.1002/cam4.3792)
Supplement: Supplementary file 1 — Table S1‐S3 [file CAM4-10-2035-s001.docx]

**SUPPORTING INFORMATION**

| **Mental Illness** | **ICD-9-CM Codes** |
| --- | --- |
| Depression | 2962X, 2963X, 3004X, 30112, 3091X, 311X |
| Bipolar disorders | 2960X; 2961X; 2964X; 2965X; 2966X; 2967X; 2968X; 2969X |
| Psychotic disorders | 29381, 29382, 295XX, 297XX, 298XX |
| Anxiety | 29384, 3000X, 30010, 3002X, 3003X, 3005X, 308XX, 30981, 3130X |
| Dementia | 2900X, 2901X, 29020, 29021, 2903X, 29040-29043, 2940X, 2941X, 3310X, 3311X, 3312X, 3317X, 797XX |
| Substance use disorders | 2910X, 2911X, 2913X - 2919X, 292XX, 303XX, 304XX, 305XX |

Abbreviations: ICD-9-CM, International Classification of Diseases, Ninth Revision, Clinical Modification.

**Supporting Table 1.** ICD-9-CM codes used to identify patients with mental illness.

| **Code Classification** | **Codes** |
| --- | --- |
| MEDPAR inpatient admission | admsrce = 7, admtype = 1 |
| HCPCS | 99281, 99282, 99283, 99284, 99285 |
| BETOS | M3 |

Abbreviations: MEDPAR, Medicare Provider Analysis and Review. HCPCS, Healthcare Common Procedure Coding System. BETOS, Berenson-Eggers Type of Service.

**Supporting Table 2.** Codes used to identify emergency department visits.

| **Code Classification** | **Codes** |
| --- | --- |
| HCPCS | 90785-90899, 96101-96103, 0359T-0374T |
| CMS specialty | 26, 27, 62, 68, 86 |
| Revenue center | 0114, 0124, 0134, 0144, 0154, 0204, 0513, 0900-0919, 0961, 1000, 1001 |

Abbreviations: HCPCS, Healthcare Common Procedure Coding System. CMS, Center for Medicare Services.

**Supporting Table 3.** Codes used to identify professional management of patients with mental illness.
